# Posterior Circulation Ischemic Stroke Secondary to High-Grade Glioma: A Rare Case Report and Review of the Literature

**DOI:** 10.7759/cureus.9824

**Published:** 2020-08-18

**Authors:** Konstantinos Kasapas, Antonia Malli, Eleni Kassioti, Polytimi-Eleni Valkimadi

**Affiliations:** 1 Neurosurgery, Athens General Hospital "G. Gennimatas", Athens, GRC; 2 Neurology, Athens Naval Hospital, Athens, GRC

**Keywords:** high grade glioma, glioblastoma, posterior cerebral artery, ischemic stroke

## Abstract

Neurological deterioration or new focal deficits in patients with primary brain tumors are usually related to intratumoral hemorrhage, disease progression, seizures (Todd paralysis) and, rarely, ischemic stroke. Ischemic strokes in this group of patients are usually a postoperative complication, a long-term result of radiation vasculopathy, embolic due to hypercoagulability and, less commonly, caused by vessel occlusion by an adjacent brain tumor. We report a rare case of ischemic stroke secondary to a newly diagnosed high-grade glioma and the possible mechanisms that resulted in this medical condition.

## Introduction

Neurological deterioration or new focal deficit in patients with primary brain tumors is usually related to intratumoral hemorrhage, disease progression, seizures (Todd paralysis) and, rarely, to ischemic stroke. Ischemic strokes in this group of patients are usually a postoperative complication, a result of radiation vasculopathy or embolic due to hypercoagulability. Furthermore, arterial ischemic stroke may be caused by vessel occlusion due to direct infiltration, encasement and occlusion of arterial branches by the adjacent brain tumor, direct tumor mass effect or leptomeningeal involvement. We report a rare case of posterior cerebral artery (PCA) stroke secondary to a newly diagnosed primary brain tumor, including a literature review on the topic.

## Case presentation

A 68-year-old man was admitted to the emergency department with sudden onset of left side muscle weakness accompanied by a four-day unsteadiness and bitemporal headache. The patient also mentioned a four-week history of dysphagia both in liquids and solids. Physical examination revealed a horizontal nystagmus in the left-eye-position, a reduced swallowing reflex, a left-sided hemiparesis with 4/5 muscle strength and a left-sided dysmetria and asynergia. His past medical history included hypertension and dyslipidemia. The rest medical history was unremarkable.

**Figure 1:**
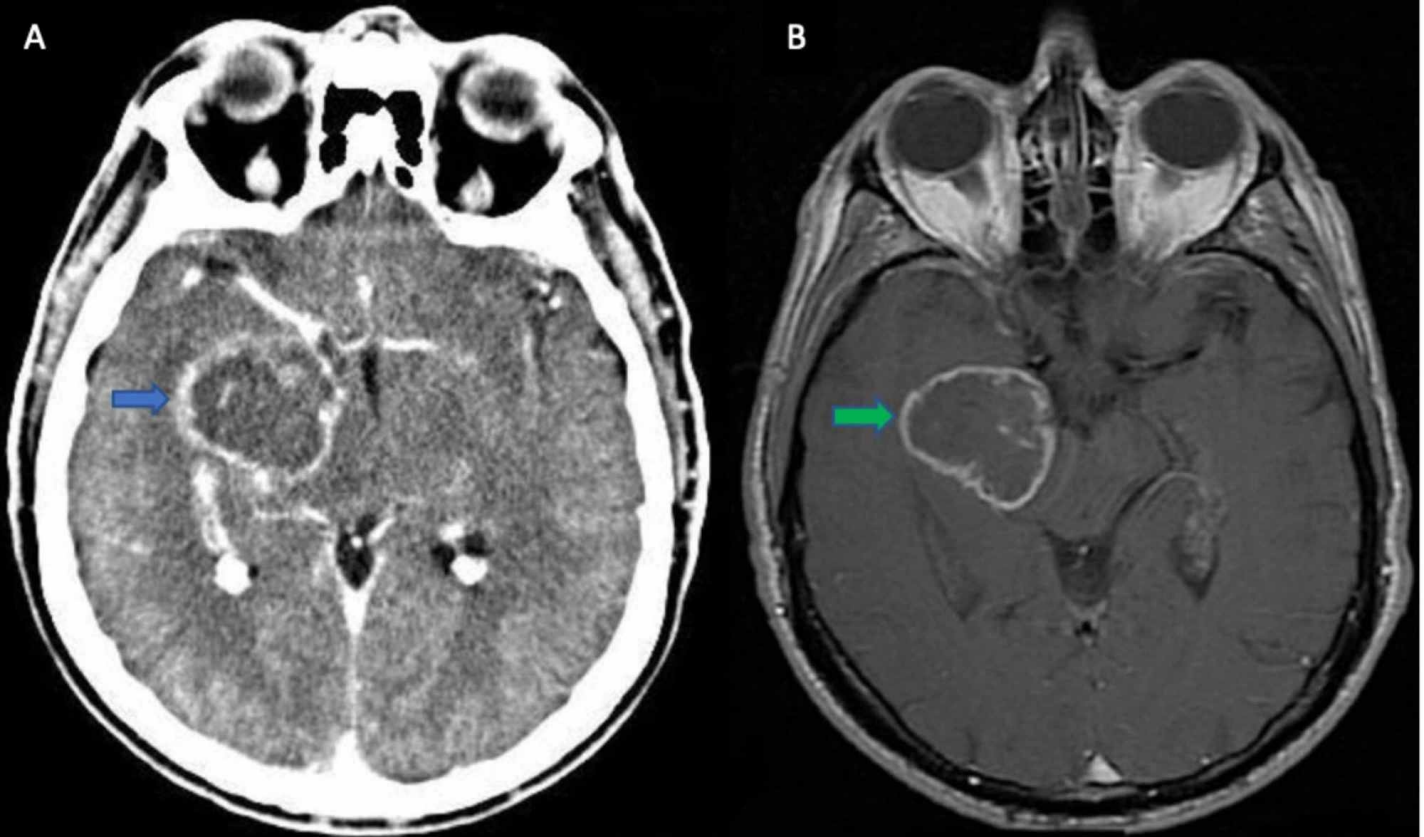
(A) CT brain axial with contrast showing a right temporal lobe tumor with an irregular ring enhancement (blue arrow). (B) MRI brain -T1 axial gadolinium (Gd) showing probably a high-grade glioma (green arrow)

The initial diagnostic workup included a brain computed tomography (CT) which revealed a right temporal lobe tumor extending into the basal ganglia as well. Further evaluation with a brain magnetic resonance imaging (MRI) revealed a highly vascularized tumor in the aforementioned area, with an irregular gadolinium ring enhancement and surrounding edema (Figure 1).

**Figure 2:**
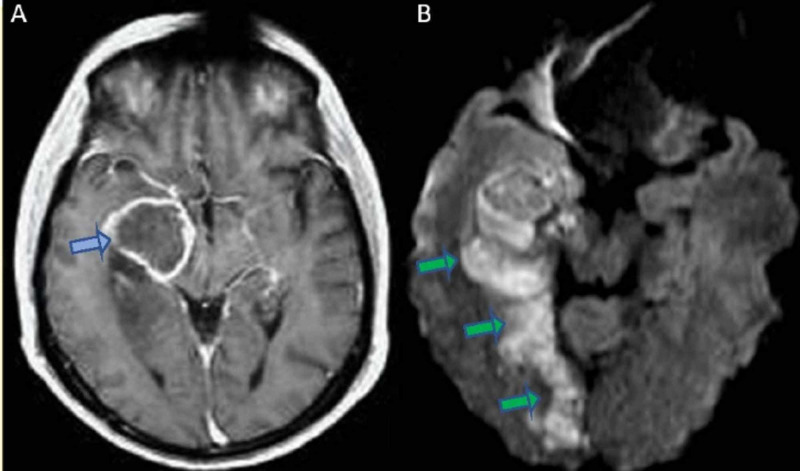
(A) MRI axial T1 gadolinium depicting a highly vascularized right temporal lobe tumor with an irregular ring enhancement (blue arrow). (B) DW-MRI scan showing a recent right posterior cerebral infarct in close proximity to the tumor (green arrows) DW-MRI: Diffusion-weighted magnetic resonance imaging

At that moment, a diagnosis of a high-grade glioma was suspected. He was treated with high dosage of intravenous dexamethasone and clinical improvement was gradually noted. Even though a primary brain lesion was most likely the case, the patient also underwent thoracic and abdomen computed tomography and the possibility of a secondary brain tumor was excluded at that stage. In further investigation, the presence of a high-grade glioma, and probably a glioblastoma, was suggested by a magnetic resonance spectroscopy (MRS). During patient’s hospitalization (day 3) a sudden neurological deterioration occurred as he developed a left hemianopia with a 2/5 left-sided hemiparesis. The new brain CT scan revealed a new hypointense lesion in the right occipital lobe without hemorrhagic findings and the diffusion-weighted magnetic resonance imaging (DW-MRI) confirmed a recent right posterior cerebral infarct (Figure 2). Thrombolysis was not performed due to high bleeding risk of the tumor and secondary stroke prevention with acetylsalicylic acid was administered at that moment. Patient’s neurological status, in terms of left-sided hemiparesis, was gradually improved and a few days later he was transferred to the neurosurgery department for surgery planning and further treatment with radiotherapy and chemotherapy.

## Discussion

In the literature, the need for “tumor awareness” in the diagnostic work-up of patients presenting with the clinical picture of acute cerebrovascular disease, has already been emphasized. However, a probably more important question is the co-existence of stroke in a patient with primary brain tumor and the relation of those two conditions, as this implies appropriate therapeutic interventions. The number of such cases is not clearly known and it is often underestimated as there are stroke cases often asymptomatic and apparent only in neuroimaging [1].

Neurological deterioration in patients with primary brain tumor usually occurs due to tumor progression (edema, necrosis), intratumoral hemorrhage, seizures or cerebral ischemia. The last cause is not that common and is often underestimated. A sudden clinical manifestation suggests a rather vascular etiology, however, the diagnosis of ischemic stroke only on clinical grounds is challenging and imaging techniques play a major role. Follow-up imaging is often necessary as DW-MRI hyperintensities around the tumor area may be related to the tumor itself or prolonged seizure activity [2].

Our literature search revealed very few cases with high-grade glioma and a posterior cerebral artery infarct, showing the rarity of such cases (Table 1). In addition, most malignant cases were associated with middle cerebral artery territories, usually following surgical resection of the tumor.

**Table 1:** Literature review for the cases of high-grade gliomas and subsequent cerebrovascular ischemic infarct MCA: Middle cerebral artery

Study	Age - Sex	Associated cerebral infarct	Management of stroke
Obeid et al. (2010) [2]	41-year-old, Male	Left middle cerebral artery infarct	Observation
Pina et al. (2014) [3]	77-year-old, Female	Right middle cerebral artery infarct	Right anterior temporal lobectomy
Rojas-Marcos et al. (2005) [4]	60-year-old, Male	Right middle cerebral artery infarct	No details
	41-year-old, Male	Right supraclinoid segment of internal carotid artery infarct	No details
Farkas et al. (2018) [5]	58-year-old, Female	Right middle cerebral artery territory infarct	Aspirin and clopidogrel – Tumor resection 2 months later
Lasocki and Gaillard (2016) [6]	58-year-old, Female	Left posterior cerebral infarct	Resection of glioblastoma
	61-year-old, Male	Right middle cerebral artery infarct	Intravenous thrombolysis with tissue plasminogen activator
Amelot et al. (2015) [7]	40-year-old, Male	Left MCA infarct	Observation
Kamiya-Matsuoka et al. (2015) [8]	Nine patients	Posterior cerebral artery infarct	No details
Chen et al. (2011) [9]	58-year-old, Female	Left middle cerebral artery infarct	Resection of glioblastoma and decompressive craniectomy

Patients with systemic cancer may develop a stroke in the context of a hypercoagulable state or nonbacterial thrombotic endocarditis (NBTE), both related to the malignancy itself. However, patients with brain tumors are not usually susceptible to these mechanisms. On the contrary, ischemic strokes in such patients are usually a postoperative complication, a late result of radiation therapy and/or chemotherapy and, less commonly, caused by vessel occlusion by direct compression, infiltration or encasement. There are also few case reports mentioning a high risk of strokes in patients receiving some certain anti-angiogenic chemotherapeutic agents such as bevacizumab [10].

In a retrospective study of 68 stroke patients with known brain tumors it was found that postoperative complications and radiation therapy accounted for 48% and 29% of stroke cases, respectively [11]. Large artery strokes accounted for 11% of nonpostoperative strokes and in the literature, other five cases of a temporal glioblastoma and a middle cerebral artery (MCA) stroke on the same side, have also been described [12-14].

Our patient was diagnosed with a right high-grade tumor and he subsequently developed a right PCA infarct a few days after the initial tumor diagnosis was made. This patient had not undergone surgical operation nor had received radiation therapy or chemotherapy at that moment. The possibility of in situ atherosclerotic disease of PCA cannot be fully excluded because of the medical history of hypertension and dyslipidemia. However, the fact that the stroke took place so soon after the initial brain tumor diagnosis and the intimate anatomical relation between the tumor and the artery, suggests a rather direct effect of the malignancy on the PCA.

There are three possible mechanisms regarding this and other relevant stroke cases with brain tumor; mechanical compression, vessel infiltration by cancer cells and local procoagulant effect due to tumor-secreted chemical factors. None of these mechanisms can be rejected from a single case report; however, a local hypercoagulant state may be more possible due to the procoagulant secreting properties of high-grade gliomas that are well-described in the current literature [15].

Intravenous thrombolysis is the only possibly effective treatment for early cerebral ischemia but the presence of an intracranial tumor has always been a contraindication because of the increased intracranial bleeding risk [16]. There are five reports so far with IV recombinant tissue plasminogen activator (r-TPA) administration in patients with intracranial neoplasms mentioning that the presence of a presumably benign neoplasm may not necessarily contribute to an unfavorable outcome and this therapeutic option should probably be kept in mind [16]. However, the number of cases is far too small and more published cases are definitely necessary [2-9].

Stroke in brain tumor patients is a frequently missed diagnosis and this leads to incomplete or even wrong evaluation and treatment. Despite this rarity, clinicians should be alert regarding this potential complication, as a false interpretation of neurological deterioration may lead to inappropriate change of antitumor therapy. In addition, while most patients have an already significant morbidity from the tumor itself, cerebral ischemia may still compromise outcome and quality of life of these patients.

Secondary prevention, when possible, should not be neglected and thrombolysis should be carefully evaluated in an individualized-patient setting due to the propensity of primary intracranial malignant neoplasms to bleed spontaneously. Last but not least, prospective studies need to be conducted to assess the treatment modalities in patients with acute stroke and a newly-diagnosed primary brain tumor.

## Conclusions

Our report describes a rare case of PCA stroke secondary to a newly diagnosed primary brain tumor. It emphasises the need of alertness for identifying other potential causes, apart from tumor progression, in such patients when clinical deterioration is observed. A precise diagnosis is necessary in order to avoid mistreating patients and help improve their outcome and quality of life. However, prospective studies need to be conducted in order to assess treatment modalities in patients with acute stroke and a newly-diagnosed primary brain tumor.
